# Supplementary material for: Predicting the risk of asthma development in youth using machine learning models
Source: PLoS One. 2025 Nov 12;20(11):e0336591. doi: 10.1371/journal.pone.0336591 (PMC12611137; doi:10.1371/journal.pone.0336591)
Supplement: S1 Table — (DOCX) [file pone.0336591.s004.docx]

**Table S1. Feature selection results by LASSO and Random Forest with absolute value of mean SHAP values calculated through the logistic regression model with undersampling.**

| **Variables selected by LASSO** | **Variables selected by Random Forest** | **Variable Description** | **Absolute Value of Mean SHAP values** |
| --- | --- | --- | --- |
| AGEP_C | AGEP_C | Age of child | 0.076407 |
| ASEV_A | ASEV_A | Adult ever had asthma | 0.011739 |
| EMERG12MTC_C | EMERG12MTC_C | Number of times visited emergency room in past 12 months | 0.003119 |
| PHSTAT_C | PHSTAT_C | General Health status | 0.042564 |
| POVRATTC_C | POVRATTC_C | Family poverty ratio | 0.000205 |
| REGION | REGION | Household region | 0.006209 |
| RX12M_C | RX12M_C | Took prescription medication in past 12 months | 0.094132 |
| SEX_C | SEX_C | Gender | 0.006066 |
| SHTFLU12M_C | SHTFLU12M_C | Received a flu shot in past 12 months | 0.002878 |
| URBRRL | URBRRL | NCHS Urban-Rural classification | 0.009671 |
| URGNT12MTC_C | URGNT12MTC_C | Number of times visited urgent care in past 12 months | 0.001943 |
| ADHDEV_C |  | Ever had ADD/ADHD | 0.008737 |
| CIGAREV_A | CIGAREV_A | Parent ever smoked a cigar | 0.004319 |
| ALCDRUGEV_C |  | Ever lived with anyone with alcohol/drug problems | 0.003006 |
| ASDEV_C |  | Ever had autism | 0.000629 |
| BNEEDS_C |  | Lifetime of lacking basic needs (Yes or No) | 0.008580 |
| COVER_C |  | Health insurance hierarchy | 0.000904 |
| DDEV_C |  | Ever had developmental delay | 0.005191 |
| DIBEV_C |  | Ever had diabetes | 0.005672 |
| ECIGEV_A |  | Parent ever used electronic cigarettes | 0.003054 |
| FDSBALANCE_C |  | How often child could not afford to eat balanced meals | 0.008007 |
| FWIC12M_C |  | Received WIC (Women, Infants, Children) benefits in past 12 months | 0.002841 |
| HISPALLP_C |  | Single and multiple race groups with hispanic origin | 0.005129 |
| LDEV_C |  | Ever had learning disability | 0.005689 |
| MEDDL12M_C |  | Delayed medical care due to cost in past 12 months | 0.004563 |
| MENTDEPEV_C |  | Ever lived with anyone mentally ill/severely depressed | 0.000030 |
| PIPEEV_A |  | Parent ever smoked a pipe filled with tobacco | 0.001613 |
| PREDIB_C |  | Ever had prediabetes | 0.001886 |
| SMKCIGST_A |  | Parent's cigarette smoking status | 0.002781 |
| TBIDAZED_C |  | Ever dazed or memory gap | 0.003726 |
| TBIHEADSYM_C |  | Ever had headache, vomit, blurred vision, or mood change after blow to head | 0.011242 |
| VIOLENEV_C |  | Victim of/witnessed violence | 0.008084 |
| WELLNESS_C |  | Was the last visit a wellness visit | 0.002786 |
| CVDSEV_C | CVDSEV_C | Symptoms of COVID-19 | 0.009377 |
