# Supplementary material for: Predicting the risk of asthma development in youth using machine learning models
Source: PLoS One. 2025 Nov 12;20(11):e0336591. doi: 10.1371/journal.pone.0336591 (PMC12611137; doi:10.1371/journal.pone.0336591)
Supplement: S2 Table — (DOCX) [file pone.0336591.s005.docx]

**Table S2. Model performance measures of machine learning models for asthma using the 2021 and 2022 NHIS undersampled data for different age groups.**

| **Age Group** | **Predictive Model** | **AUC score** | **Precision** | **Recall** | **Accuracy** | **F1 Score** |
| --- | --- | --- | --- | --- | --- | --- |
| **Age 0-4  (n = 1,808)** | **Random Forest** | 0.7262 | 0.0783 | 0.6500 | 0.7053 | 0.1398 |
|  | **XGBoost** | 0.6557 | 0.0591 | 0.6500 | 0.6059 | 0.1083 |
|  | **Neural Network** | 0.6305 | 0.0741 | 0.5000 | 0.7514 | 0.1290 |
|  | **Logistic Regression** | 0.6464 | 0.0657 | 0.6500 | 0.6464 | 0.1193 |
|  | **SVM (linear)** | 0.6154 | 0.0579 | 0.5500 | 0.6538 | 0.1048 |
| **Age 5-12**  **(n = 4,582)** | **Random Forest** | 0.7356 | 0.2137 | 0.6732 | 0.6880 | 0.3244 |
|  | **XGBoost** | 0.6921 | 0.1813 | 0.6340 | 0.6407 | 0.2820 |
|  | **Neural Network** | 0.6661 | 0.2644 | 0.5098 | 0.7876 | 0.3482 |
|  | **Logistic Regression** | 0.7451 | 0.2240 | 0.6471 | 0.7113 | 0.3328 |
|  | **SVM (linear)** | 0.7488 | 0.2276 | 0.6471 | 0.7164 | 0.3367 |
| **Age 13-17**  **(n = 3,326)** | **Random Forest** | 0.6728 | 0.2033 | 0.6250 | 0.6152 | 0.3069 |
|  | **XGBoost** | 0.6413 | 0.1976 | 0.6030 | 0.6122 | 0.2976 |
|  | **Neural Network** | 0.6152 | 0.2555 | 0.4265 | 0.7525 | 0.3196 |
|  | **Logistic Regression** | 0.6905 | 0.2330 | 0.5809 | 0.6824 | 0.3326 |
|  | **SVM (linear)** | 0.6860 | 0.2235 | 0.5588 | 0.6754 | 0.3193 |
