# Supplementary material for: Predicting the risk of asthma development in youth using machine learning models
Source: PLoS One. 2025 Nov 12;20(11):e0336591. doi: 10.1371/journal.pone.0336591 (PMC12611137; doi:10.1371/journal.pone.0336591)
Supplement: S3 Table — (DOCX) [file pone.0336591.s006.docx]

**Table S3. External validation of machine learning models for asthma using the 2023 NHIS undersampled data for different age groups.**

| **Age Group** | **Predictive Model** | **AUC score** | **Precision** | **Recall** | **Accuracy** | **F1 Score** |
| --- | --- | --- | --- | --- | --- | --- |
| **Age 0-4 (n = 853)** | **Random Forest** | 0.6970 | 0.0850 | 0.6744 | 0.6178 | 0.1510 |
|  | **XGBoost** | 0.6518 | 0.0840 | 0.7209 | 0.5897 | 0.1505 |
|  | **Neural Network** | 0.5446 | 0.0642 | 0.3953 | 0.6788 | 0.1104 |
|  | **Logistic Regression** | 0.6552 | 0.0781 | 0.6977 | 0.5698 | 0.1405 |
|  | **SVM (linear)** | 0.6270 | 0.0782 | 0.6512 | 0.5955 | 0.1397 |
| **Age 5-12**  **(n = 1,680)** | **Random Forest** | 0.7134 | 0.2117 | 0.7243 | 0.6214 | 0.3277 |
|  | **XGBoost** | 0.6809 | 0.1906 | 0.7009 | 0.5827 | 0.3000 |
|  | **Neural Network** | 0.6433 | 0.2201 | 0.5935 | 0.6804 | 0.3211 |
|  | **Logistic Regression** | 0.7120 | 0.1898 | 0.7336 | 0.5673 | 0.3016 |
|  | **SVM (linear)** | 0.7100 | 0.2029 | 0.7243 | 0.6024 | 0.3170 |
| **Age 13-17**  **(n = 1,710)** | **Random Forest** | 0.6874 | 0.2283 | 0.6477 | 0.6076 | 0.3376 |
|  | **XGBoost** | 0.6085 | 0.2077 | 0.5530 | 0.6053 | 0.3020 |
|  | **Neural Network** | 0.6257 | 0.2767 | 0.4811 | 0.7257 | 0.3513 |
|  | **Logistic Regression** | 0.6887 | 0.2355 | 0.6174 | 0.6316 | 0.3410 |
|  | **SVM (linear)** | 0.6726 | 0.2354 | 0.5795 | 0.6444 | 0.3348 |
